# Continuity corrections with Mantel–Haenszel estimators in Cochrane reviews

**DOI:** 10.1017/rsm.2025.10012

**Published:** 2025-06-06

**Authors:** A.E. Ades, Deborah M. Caldwell, Sumayya Anwer, Sofia Dias

**Affiliations:** 1 Population Health Sciences, University of Bristol Medical School, Bristol, UK; 2 Centre for Reviews and Dissemination, University of York, York, UK

Dear Editors

The paper by Tsujimoto et al.^1^ raises serious questions about the conduct of Cochrane reviews. They found that 64% of 885 meta-analyses that included studies with zero cells implemented the Mantel–Haenszel (MH) method in RevMan software, which by default implements the standard continuity correction (CC), in which 0.5 is added to every cell of a 2 × 2 table with a zero cell. The CC is unnecessary when calculating MH statistics, and it biases estimates towards the null. Tsujimoto et al. reported that in about 30% of cases, point estimates of the odds ratio were biased by 25% or more.

Readers will surely support their conclusion that ‘future updates of RevMan should incorporate less biased methods,’ and their call for MH without CC to be incorporated. However, ‘bias’ may be too mild a word for a procedure that is, and has always been, recognized as frankly *incorrect.* ^2^ Readers might have expected a call for biased methods to be entirely removed: they might also be wondering how the Cochrane organization could have allowed this blunder to persist for so long.

In 2016, we raised RevMan’s CC issue with the Cochrane Neonatal group, following the 2014 publication of their review of intrapartum anti-bacterial prophylaxis (IAP) to prevent neonatal early-onset Group B streptococcus (EOGBS) infection.^3^ The headline estimate (0.17, 95%CI 0.04–0.74) of the efficacy of IAP to prevent EOGBS disease included a CC. This underestimates the efficacy by a factor of nearly 2 (Table 1). Our communication with the Neonatal Group and the authors’ response was recorded as ‘Feedback’ on pages 32 and 33 of the Cochrane review.

**Table 1:** Efficacy of IAP in Cochrane reviews, published estimates compared to MH odds ratios without continuity correction (CC).^8^

Cochrane review	Number of trials	Outcome	Method	Cochrane estimate	MH estimate without CC
Smaill (2000)^6^	4	Infant GBS colonization	Peto OR	0.10 (0.07–0.14)	0.037 (0.018–0.074)
Smaill (2000)^6^	4	EOGBS disease	Peto OR	0.17 (0.07–0.39)	0.051 (0.007–0.375)
Ohlsson (2014)^3^	3	EOGBS disease	MH RR with CC^a^	0.17 (0.04–0.74)	0.097 (0.014–0.70)

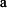
 Outcomes are sufficiently rare that relative risks and odds ratios are effectively equivalent.

The authors replied: ‘We are not statisticians, but assume that the statistical methods used in RevMan 5.2 are correct’. They went on to quote the 2008 Cochrane Handbook section 9.2.2.2 to the effect that ORs and RR cannot be calculated if there are zero cells, and that RevMan ‘automatically makes the [continuity] correction when necessary’.^4^ Almost identical wording appears in section 6.4.1.2 of the 2nd edition.^5^

The author’s response is understandable, and it is hard to attach any blame to the distinguished neonatologist at Toronto’s Hospital for Sick Children who led the review. Of course, a later section (16.9 in the 2008 Handbook, 10.4.4.1 in the 2019 edition) explains that the correction is not necessary when the MH estimators are used, and also that it causes bias. However, even if the Handbook had given explicit advice *not* to implement the CC with MH estimators, this would have made no difference: until recently, the use of RevMan was mandatory in Cochrane reviews (Handbook section 2.3.5).

An earlier version of the IAP Cochrane review^6^ had used the Peto method, another inappropriate choice, as it biases towards the null when the treatment effect is strong (Table 1).^7^ This suggests that the absence of effective statistical oversight had been a long-standing problem. According to our estimates, based on a synthesis of both EOGBS colonization and EOGBS disease outcomes, the 2014 review under-estimated the effect on GBS disease by a factor of more than 5.^8^ Errors of this magnitude have profound public health implications: while the efficacy of IAP is not in question, the question of how it should be delivered, following screening or to high-risk groups, is still being actively researched.

The authors of the 2014 review noted that there was a high risk of bias in the three trials reporting EOGBS as an outcome. They concluded: ‘There is a lack of evidence from well-designed and conducted trials to recommend IAP to reduce the risk of EOGBS disease’. One has to be concerned that their underestimation of the efficacy of IAP may have influenced this judgement.

Although their conclusions may be technically justified, they were—and still are—at variance with previously published meta-analyses (not using a CC),^9^ ^–^ ^11^ and entirely contrary to clinical policy and practice in most developed countries, both at the time and now, and indeed in the hospital where they worked. In both the USA^12^ and Canada^13^ IAP was first recommended in official guidance over 25 years ago, *its efficacy premised on the same trials examined in the Cochrane reviews*. IAP interventions of one form or another have been recommended in every update since. In a 2007 position statement, the Canadian Paediatric Society declared that IAP was ‘highly effective’ in preventing EOGBS.^14^ By 2017, 60 countries out of 95 surveyed operated some form of IAP policy.^15^

MH with no CC is standard in *metafor* software,^16^ ^,^ ^17^ and is available in STATA^18^ and the R *meta* package.^19^ ^,^ ^20^ There are alternative CCs besides the standard 0.5. Zabriskie et al.^21^ comprehensively review previous work on meta-analysis with zero cells, and document new simulations of MH and other pooling methods paired with alternative CCs. Some of these perform better with MH pooling than MH with no CC and are available in STATA and R *meta*.

Possibly, if the 2014 Cochrane review was repeated now, the outcome would be different. The recent Methodological Expectations in Cochrane Reviews guidance^22^ incorporates PRISMA 2020, which includes (Item 23a) a requirement to ‘provide a general interpretation of the results in the context of other evidence’.^23^ This would have obliged authors to ask why previous reviews of essentially the same evidence had produced different results. Other changes in the Cochrane organization, including the more centralized editorial process, might be expected to result in a more effective oversight of statistical methods.

Such optimism may however be misplaced. The standard 0.5 CC with MH pooling is still the default in STATA and is obligatory in both Comprehensive Meta-analysis (CMA)^24^ and RevMan.

We approached the Cochrane Support website and asked whether the RevMan technical team was planning to make any changes, such as (1) providing a version of MH without the CC, (2) making this the default option, or (3) removing the version with the CC. The response, forwarded from the Methods and Synthesis Development Team, was that ‘there are no planned changes to MH Calculations,’ that ‘any changes to statistical approaches are based on recommendations from the Statistics Methods Group,’ and that ‘their advice on this issue had not changed over the years’.

## Data Availability

None declared.
